# Plasminogen Activator Inhibitor-2 Plays a Leading Prognostic Role among Protease Families in Non-Small Cell Lung Cancer

**DOI:** 10.1371/journal.pone.0133411

**Published:** 2015-07-31

**Authors:** Chia-Yi Su, Yu-Peng Liu, Chih-Jen Yang, Yuan-Feng Lin, Jean Chiou, Li-Hsing Chi, Jih-Jong Lee, Alex T. H. Wu, Pei-Jung Lu, Ming-Shyan Huang, Michael Hsiao

**Affiliations:** 1 Genomics Research Center, Academia Sinica, Taipei, Taiwan; 2 Department of Genome Medicine, Kaohsiung Medical University, Kaohsiung, Taiwan; 3 Department of Internal Medicine, Kaohsiung Medical University Hospital, Kaohsiung Medical University, Kaohsiung, Taiwan; 4 Graduate Institute of Clinical Medicine, College of Medicine, Taipei Medical University, Taipei, Taiwan; 5 The Ph.D. Program for Cancer Biology and Drug Discovery, China Medical University and Academia Sinica, Taichung, Taiwan; 6 The Ph.D. Program for Translational Medicine, Taipei Medical University and Academia Sinica, Taipei, Taiwan; 7 Institute of Veterinary Clinical Science, School of Veterinary Medicine, National Taiwan University, Taipei, Taiwan; 8 Institute of Clinical Medicine, National Cheng-Kung University, Tainan, Taiwan; University of Pécs Medical School, HUNGARY

## Abstract

**Background:**

In lung cancer, uPA, its receptor (uPAR), and the inhibitors PAI-1 and PAI-2 of the plasminogen activator family interact with MMP-2 and MMP-9 of the MMP family to promote cancer progression. However, it remains undetermined which of these markers plays the most important role and may be the most useful indicator to stratify the patients by risk.

**Methods:**

We determined the individual prognostic value of these 6 markers by analyzing a derivation cohort with 98 non-small cell lung cancer patients by immunohistochemical staining. The correlation between the IHC expression levels of these markers and disease prognosis was investigated, and an immunohistochemical panel for prognostic prediction was subsequently generated through prognostic model analysis. The value of the immunohistochemical panel was then verified by a validation cohort with 91 lung cancer patients.

**Results:**

In derivation cohort, PAI-2 is the most powerful prognostic factor (HR = 2.30; *P* = 0.001), followed by MMP-9 (HR = 2.09; *P* = 0.019) according to multivariate analysis. When combining PAI-2 and MMP-9, the most unfavorable prognostic group (low PAI-2 and high MMP-9 IHC expression levels) showed a 6.40-fold increased risk of a poor prognosis compared to the most favorable prognostic group (high PAI-2 and low MMP-9 IHC expression levels). PAI-2 and MMP-9 IHC panel could more precisely identify high risk patients in both derivation and validation cohort.

**Conclusions:**

We revealed PAI-2 as the most powerful prognostic marker among PA and MMP protease family even after considering their close relationships with each other. By utilizing a combination of PAI-2 and MMP-9, more precise prognostic information than merely using pathological stage alone can be obtained for lung cancer patients.

## Introduction

Lung cancer remains the most dominant cause of cancer-related death in spite of advances in treatment strategies [1]. Stage-based management were generally used as the guide to decide which patients should receive surgery, chemotherapy, or radiotherapy, or targeted therapy [2]. However, different prognosis was not uncommonly seen in patients within the same stage. Although high risk factors such as poorly differentiated tumors and vascular or pleural involvement were recommended to be used as additional indicators for more aggressive treatment, these risk factors alone are still not enough to precisely stratify the patients by risk [3,4]. Therefore, more novel prognostic predictive markers are urgently needed to guide therapeutic decision-making. Recently, studies that compare and combine markers of cancer regulatory pathways, such as the pathways controlling tumor proliferation [5] and the epithelial-mesenchymal transition [6], with the aim of generating greater prognostic efficacy to identify high risk patients have received increasing attention.

In lung cancer, the high incidence of local aggressiveness and metastatic behavior is one of the main causes of cancer-related mortality and may lead to treatment failure [7]. Extracellular matrix (ECM) degradation is one of the most crucial steps involved in local invasion and distant metastasis [8]. The plasminogen activator (PA) family and the matrix metalloproteinase (MMP) family are two well-known protease families that play essential roles in ECM degradation during cancer progression [9,10], and their members have been reported to be useful prognostic markers in lung cancer. High expression levels of urokinase-type plasminogen activator (uPA), its receptor (uPAR), and plasminogen activator inhibitor type 1 (PAI-1) in the PA family and matrix metalloproteinase 2 (MMP-2) and matrix metalloproteinase 9 (MMP-9) in the MMP family have been correlated with a poor prognosis and unfavorable clinicopathological parameters [11–18]. In contrast, patients with high plasminogen activator inhibitor type 2 (PAI-2) expression levels tend to have a favorable prognosis [19–21]. Although in some cancer types such as endometrial and colorectal cancers, high PAI-2 expression was associated with poor prognosis [22,23], high PAI-2 expression was also shown to be correlated with better prognosis in breast and ovarian cancer [24,25].

In addition to acting alone, molecules in the PA and MMP families also interact with each other to further modulate the process of ECM degradation in a complicated manner (S1 Fig). Plasmin, which is activated from plasminogen by the binding of uPA to uPAR, can degrade the ECM directly or indirectly through the proteolytic activation of MMP-2 and MMP-9 [26]. When either PAI-1 or PAI-2 is present, the ability of uPA to activate plasmin is inhibited, and in turn, ECM degradation is also inhibited. However, uPA-PAI-1 complexes have also been reported to increase MMP-2 and MMP-9 expression level through downstream signaling pathway [27], whereas the uPA-PAI-2 complex has been shown to facilitate the clearance of uPA without activating downstream signaling [28]. Moreover, MMP-9 can enhance uPA activity by regulating protease nexin-1 cleavage [29]. These markers form a complex network that regulates the balance of ECM degradation in the tumor microenvironment.

Therefore, identification of the most important markers within the signaling network for prognostic and therapeutic decision will increase the clinical value. Rather than focusing on a single marker, our primary interest in this study was to assess and compare the prognostic value of markers in PA and MMP families and to evaluate their combined effects. After immunohistochemical staining of clinical non-small cell lung cancer (NSCLC) specimens to determine the expression levels of the components of this network, a three-step approach was used to integrate the prognostic values of uPA, uPAR, PAI-1, PAI-2, MMP-2, and MMP-9. We first verified the correlation between these 6 markers and then carried out a multi-marker assessment, whereby we identified PAI-2 as the marker with the greatest prognostic value and MMP-9 as the second most powerful prognostic indicator. Finally, a practical IHC panel composed of PAI-2 and MMP-9 was generated for improved prognostic utility.

## Materials and Methods

### Ethics statement and Patients

The study was carried out with the approval of the Institutional Review Boards and with the permission from the ethics committees of the institution involved (KMUH-IRB-20110286), Institutional Review Board of Kaohsiung Medical University Chung-Ho Memorial Hospital. No informed consent was required because the data were analyzed anonymously. No patients' treatments were modified for the purpose of this study.

A total of 98 NSCLC patients, including 61 cases of adenocarcinoma, 31 cases of squamous cell carcinoma, and 6 cases of large cell carcinoma, from Kaohsiung Medical University Hospital of Taiwan from 1991 to 2007 were enrolled as derivation cohort (S1 Table). All patients received standard treatment protocols according to hospital guidelines. Patients with operable stage I-III NSCLC underwent lobectomy or pneumonectomy with mediastinal lymphadenectomy. Patients with resectable stage II and III NSCLC were treated with postoperative adjuvant platinum-based chemotherapy. Patients with nonresectable locally advanced or metastatic disease received chemotherapy with or without radiotherapy. Representative 1-mm-diameter cores from each tumor with morphological features that were typical of the diagnosis were obtained from formalin-fixed paraffin embedded tissue to generate a tissue microarray (TMA). Overall survival (OS) and disease-free survival (DFS) were defined as the intervals from the initial treatment time until death or until recurrence, metastasis, or death, respectively. All cases were staged according to the American Joint Committee on Cancer staging manual, and the histological cancer types were classified according to the criteria of the World Health Organization classification. Another non-small cell lung cancer TMA with 91 cases was purchased from SuperBioChips (CC5 and CCA4, SuperBioChips Laboratories, Seoul, Korea) as the validation cohort (S1 Table).

### Immunohistochemical (IHC) staining and interpretation

IHC staining for uPA, uPAR, PAI-1, PAI-2, MMP-2, and MMP-9 was performed on serial 5-micrometer-thick tissue sections cut from the tissue microarray (TMA). Before staining on the TMA of derivation and validation cohort, we tested and titrated the primary antibody on a test-TMA which contains 6 normal lung tissue and 6 NSCLC tissue. A staining condition which we can discriminate the staining intensity between cases was considered as an appropriate staining condition. After determine the staining condition for all markers, IHC staining was performed using an automated immunostainer (Ventana Discovery XT autostainer, Ventana, USA) for uPA, uPAR, PAI-1, PAI-2, and MMP-9. The slides were stained with a polyclonal rabbit anti-PAI-1 antibody (sc-8979, 1:50; Santa Cruz Biotechnology, Santa Cruz, CA), a polyclonal rabbit anti-PAI-2 antibody (16035-1-AP, 1:50; Proteintech, Chicago, USA), a polyclonal rabbit anti-uPA antibody (sc-14019, 1:50; Santa Cruz Biotechnology, Santa Cruz, CA), a polyclonal rabbit anti-uPAR antibody (GTX100466, 1:750; GeneTex, Taipei, Taiwan), and a polyclonal rabbit anti-MMP-9 antibody (#2270, 1:50; Cell Signaling Technology, Danvers, MA, USA). Manual IHC staining was performed for MMP-2. Briefly, the slides were subjected to heat-induced antigen retrieval for 10 minutes with DAKO antigen retrieval buffer (pH 6) and incubated at 4°C overnight with a polyclonal rabbit anti-MMP-2 antibody (10373-2-AP, 1:50; Proteintech, Chicago, USA) and then visualized using a 3, 3’-diaminobenzidine (DAB) peroxidase substrate kit (Vector Laboratories, USA). Negative control was performed by replacing the primary antibody by a rabbit polyclonal IgG isotype control (ab27478, 1:100; Abcam, Cambridge, UK) (S2 Fig).

IHC staining was independently analyzed by 2 pathologists (Chia-Yi Su and Michael Hsiao) who were blinded to the patient’s outcome. We adapted the IHC scoring methods from previous studies to establish a uniform IHC scoring criteria for all 6 markers. Like the scoring system used by previous research focused on PA family [20] and MMP-2 and MMP-9 [17], both the cytoplasmic staining intensity and the percentage (0–100%) of tumor cells stained were recorded in present study. The staining intensity was scored using a four-tier scale: 0, negative; 1+, weak; 2+, moderate; 3+, strong. High IHC expression level was defined as a staining intensity of 2+ or 3+ in more than 25% of the tumor cells, and the other cases were considered as having low IHC expression levels (S3 Fig).

### Statistical analysis

The statistical analysis was performed using SPSS 17.0 software (SPSS, USA). Correlations for IHC expression level of all 6 markers were evaluated using Spearman’s rank correlation analysis. The survival rates were estimated using the Kaplan-Meier survival analysis and univariate and multivariate Cox regression analyses. For the analysis with multiple comparisons, Benjamini and Hochberg false discovery rate (FDR)-corrected *P* value was used, and a FDR-corrected *P* value ≤ 0.05 was considered significant [30]. For the prognostic model analysis, a more complex model was generated by adding each marker sequentially into the former prognostic model to determine whether the added marker significantly improved the prognostic prediction. The difference between models was also compared by the area under the curve (AUC) of ROC (receiver operative characteristic) curve, sensitivity, and specificity. For all analyses, a *P* value <0.05 was regarded as statistically significant.

## Results

### Verification of the correlation between IHC expression levels of uPA, uPAR, PAI-1, PAI-2, MMP-2, and MMP-9 in NSCLC

**Fig 1:**
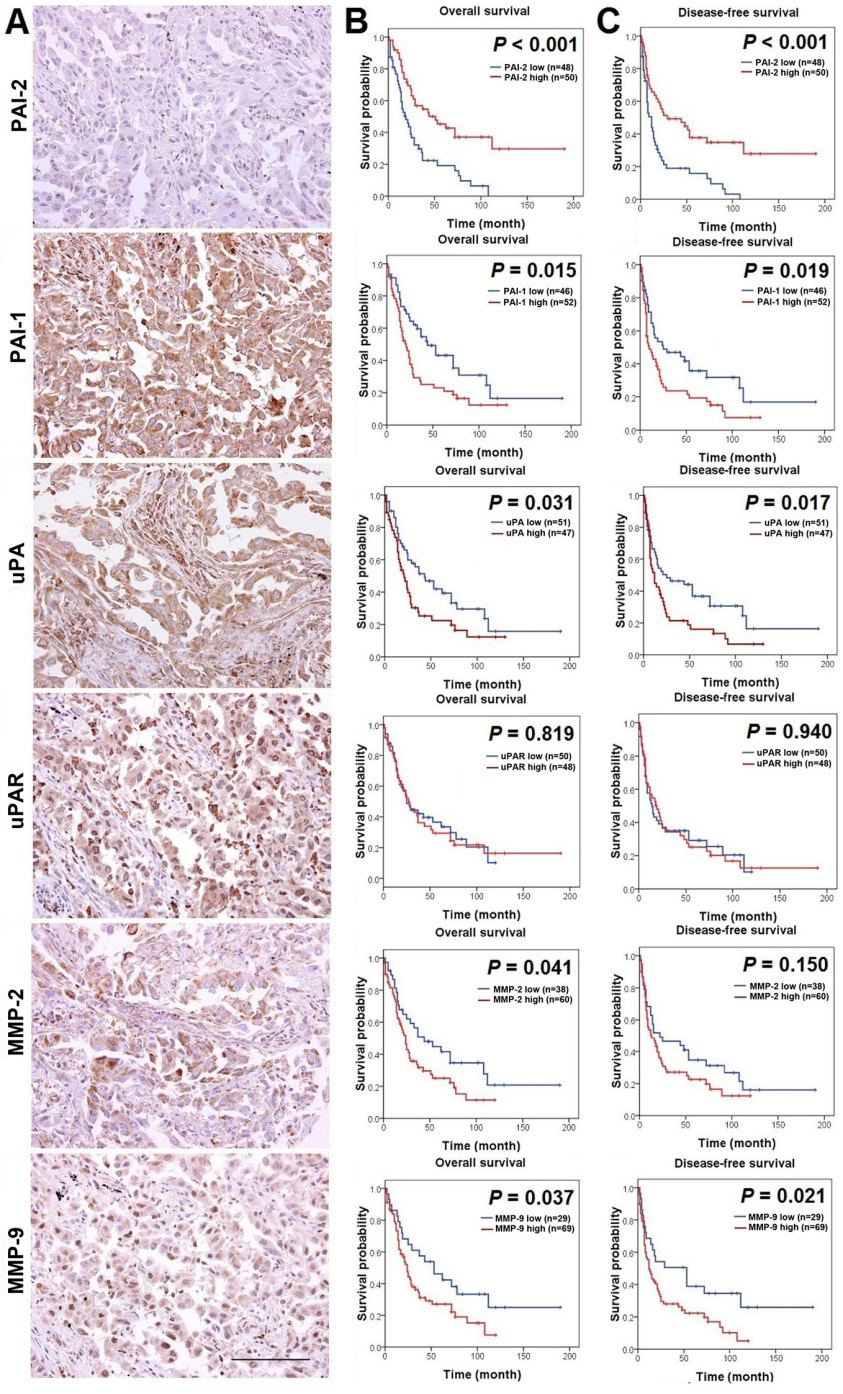
The prognostic value of markers in PA and MMP families in derivation cohort with 98 NSCLC cases. (A) A representative lung adenocarcinoma with low IHC expression level of PAI-2 and high IHC expression levels of PAI-1, uPA, uPAR, MMP-2, and MMP-9, as detected by IHC. Photographs were taken at a magnification of 400×. Scale bars represent 100 μm. (B) Kaplan-Meier plots of overall survival relative to PAI-1, PAI-2, uPA, uPAR, MMP-2, and MMP-9 IHC expression levels in NSCLC. Low IHC expression level of PAI-2 was significantly associated with shorter overall survival (*P* < 0.001), as was high IHC expression levels of PAI-1, uPA, MMP-2, and MMP-9 (*P* = 0.015, *P* = 0.031, *P* = 0.041, *P* = 0.037, respectively). (C) Kaplan-Meier plots of disease-free survival relative to PAI-1, PAI-2, uPA, uPAR, MMP-2, and MMP-9 IHC expression levels in NSCLC. Low IHC expression level of PAI-2 was significantly associated with shorter disease-free survival (*P* < 0.001); high IHC expression levels of PAI-1, uPA, and MMP-9 was also significantly associated with shorter disease-free survival (*P* = 0.019, *P* = 0.017, *P* = 0.021, respectively).

First, in order to verify whether there are significant correlations between the IHC expression status of uPA, uPAR, PAI-1, PAI-2, MMP-2, and MMP-9, Spearman’s rank correlation analysis was performed in our derivation cohort. The results identified significant correlations (*P* < 0.01) between PAI-1 and uPA and uPAR IHC expression levels (ρ = 0.616 and 0.431, respectively), between uPA and uPAR IHC expression levels (ρ = 0.571), between MMP-9 and uPA and uPAR IHC expression levels (ρ = 0.354 and 0.322, respectively), and between MMP-2 and uPA IHC expression levels (ρ = 0.261). A significant inverse correlation was observed between IHC expression levels of PAI-2 and uPA (ρ = -0.246; *P* = 0.015), and PAI-2 IHC expression level also tended to be negatively correlated with IHC expression levels of other markers (Table 1). The IHC images shown in Fig 1A represent serial sections of a lung adenocarcinoma with low PAI-2 staining intensity and high staining intensity of all 5 of the other markers.

**Table 1:** The correlations between IHC expression levels of PAI-1, PAI-2, uPA, uPAR, MMP-2, and MMP-9 analyzed by Spearman’s rank correlation analysis in derivation cohort with 98 NSCLC cases.

	PAI-2	PAI-1	uPA	uPAR	MMP-2	MMP-9
PAI-2	1					
PAI-1	-0.142	1				
uPA	-0.246*	0.616**	1			
uPAR	-0.062	0.431**	0.571**	1		
MMP-2	-0.142	0.175	0.261**	0.109	1	
MMP-9	-0.080	0.197	0.354**	0.322**	0.126	1

**Correlation is significant at the 0.01 level (2-tailed).

*Correlation is significant at the 0.05 level (2-tailed).

PAI-1: plasminogen activator inhibitor-1; PAI-2: plasminogen activator inhibitor-2; uPA: urokinase-type plasminogen activator; uPAR: urokinase-type plasminogen activator receptor; MMP-2: matrix metalloproteinase 2; MMP-9: matrix metalloproteinase 9.

### Determination of the greatest prognostic significance of PAI-2 and MMP-9 among PA and MMP family markers

To determine the individual prognostic power of these 6 markers, we first evaluated the association between their IHC expression levels and patient outcomes by Kaplan-Meier survival analysis in the derivation cohort. Low PAI-2 IHC expression level was significantly correlated with a poor prognosis (*P* < 0.001 for both overall survival (OS) and disease-free survival (DFS)) (Fig 1B and 1C). High IHC expression level of PAI-1, uPA, and MMP-9 also correlated with a poor prognosis. High MMP-2 IHC expression level was associated with an unfavorable OS but was not associated with DFS. The uPAR IHC expression level was not correlated with either OS or DFS. Besides, through clinicopathological analysis, a significant correlation between low PAI-2 IHC expression level and higher pathological stage (*P* = 0.027) was found (S1 Table and S4 Fig). On the contrary, high IHC expression levels of other markers tended to have a correlation with higher pathological stage. The correlations between each marker and other clinicopathological characteristics are summarized in S1 Table.

Due to the significant correlation between each marker in the correlation analysis, a multivariate analysis was performed to determine their respective prognostic roles (Table 2). For DFS, low PAI-2 IHC expression level (hazard ratio [HR] = 2.30; 95% confidence interval [CI] = 1.40–3.79; *P* = 0.001), high MMP-9 IHC expression level (HR = 2.09; 95% CI = 1.13–3.88; *P* = 0.019), and a higher pathological stage (HR = 3.44; 95% CI = 2.03–5.84; *P* < 0.001) remained independently correlated with an unfavorable prognosis. With regard to OS, only PAI-2 IHC expression level (HR = 2.52; 95% CI = 1.50–4.22; *P* < 0.001) and pathological stage (HR = 4.14; 95% CI = 2.39–7.16; *P* < 0.001) retained a significant influence. After false discovery rate correction for multiple testing, PAI-2 (*P* = 0.01) and MMP-9 (*P* = 0.05) still had prognostic significance in DFS. These results indicated that PAI-2 has the leading prognostic value and MMP-9 is the second most significant prognostic marker among multiple markers in PA and MMP families.

**Table 2:** Cox multivariate analysis with false discovery rate correction of PAI-1, PAI-2, uPA, uPAR, MMP-2 and MMP-9 IHC expression levels and pathological stage in derivation cohort with 98 NSCLC cases.

	Disease-free survival			Overall survival		
Variables	HR (95%CI)	*P*	FDR-corrected *P*-value*	HR (95%CI)	*P*	FDR-corrected *P*-value*
Pathological stage						
I-II	1			1		
III-IV	3.44 (2.03–5.84)	<0.001	0.01	4.14 (2.39–7.16)	<0.001	0.01
PAI-2						
High	1			1		
Low	2.30 (1.40–3.79)	0.001	0.01	2.52 (1.50–4.22)	<0.001	0.01
PAI-1						
Low	1			1		
High	1.69 (0.91–3.13)	0.099	0.18	1.67 (0.88–3.17)	0.115	0.21
uA						
Low	1			1		
High	1.06 (0.54–2.11)	0.863	0.88	0.86 (0.43–1.71)	0.668	0.67
uPAR						
Low	1			1		
High	0.65 (0.37–1.16)	0.653	0.88	0.87 (0.50–1.51)	0.611	0.67
MMP-2						
Low	1			1		
High	1.08 (0.64–1.82)	0.774	0.88	1.60 (0.92–2.80)	0.098	0.21
MMP-9						
Low	1			1		
High	2.09 (1.13–3.88)	0.019	0.05	1.49 (0.79–2.80)	0.218	0.31

*P value after Benjamini and Hochberg false discovery rate (FDR) procedure.

PAI-1: plasminogen activator inhibitor-1; PAI-2: plasminogen activator inhibitor-2; uPA: urokinase-type plasminogen activator; uPAR: urokinase-type plasminogen activator receptor; MMP-2: matrix metalloproteinase 2; MMP-9: matrix metalloproteinase 9.

### Establishment of an IHC panel comprising PAI-2 and MMP-9 for more precise prognostic prediction in NSCLC patients

**Fig 2:**
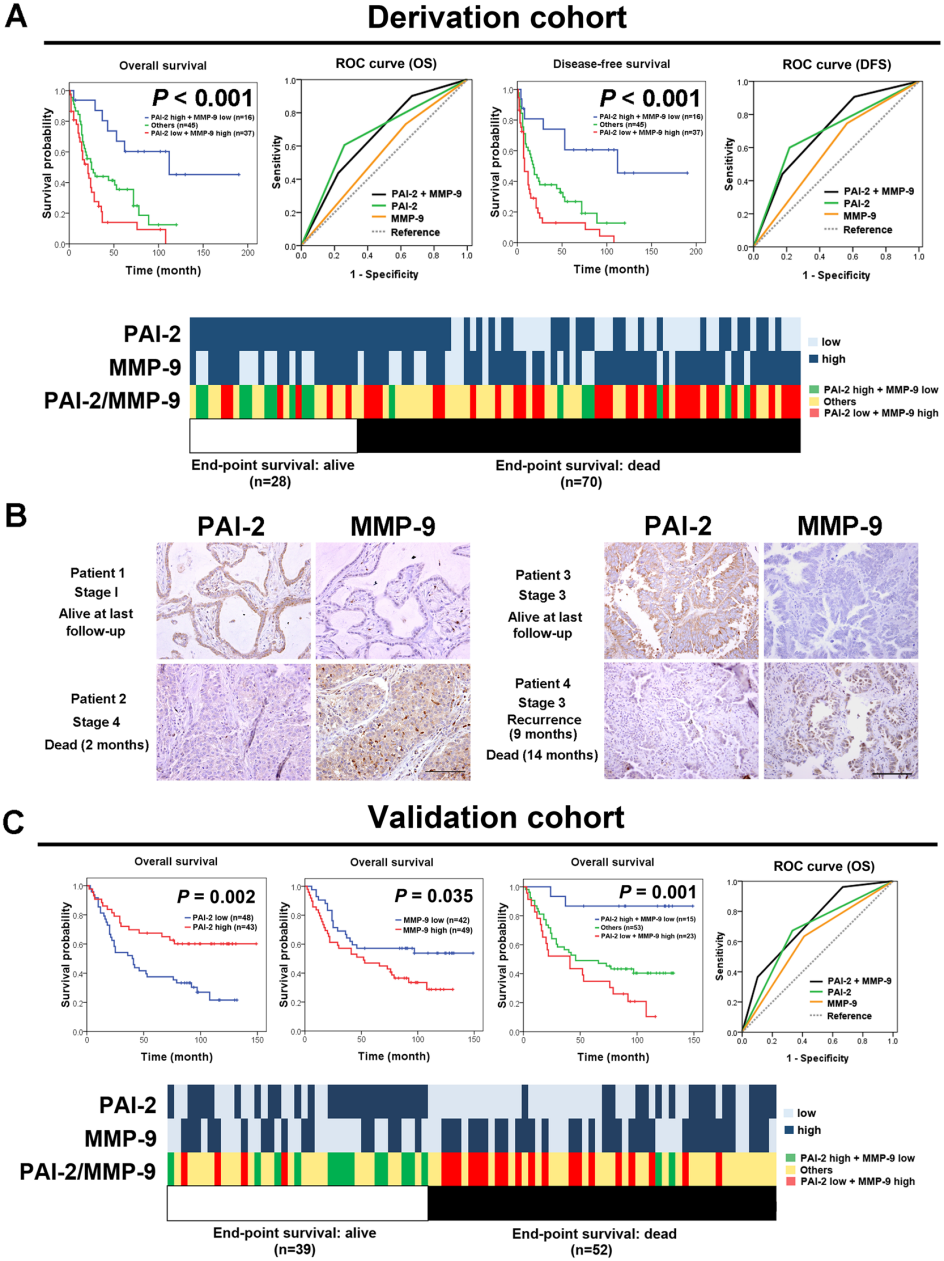
An IHC panel comprising PAI-2 and MMP-9 provides a more precise prognostic predictive power for 98 NSCLC patients in derivation cohort and 91 NSCLC patients in validation cohort. (A) Kaplan-Meier plots and ROC curve of overall survival and disease-free survival for the combination of PAI-2 and MMP-9 as an IHC panel. Patients with high PAI-2 and low MMP-9 IHC expression levels had the most favorable prognosis, whereas patients with low PAI-2 and high MMP-9 IHC expression levels had the most unfavorable prognosis (*P* < 0.001 for both OS and DFS). Increase of area under curve (AUC) of ROC was observed after combining PAI-2 and MMP-9 as a panel in disease-free survival. In the heat map clustered by end-point survival status, patients who were dead at the end-point tended to have low PAI-2 and high MMP-9 IHC expression level. (B) Representative images of the usage of the PAI-2 and MMP-9 IHC panel in clinical NSCLC samples. Patient 1 had stage I adenocarcinoma with high PAI-2 and low MMP-9 IHC expression levels and was alive at the final follow up. In contrast, patient 2 had stage IV adenocarcinoma with low PAI-2 and high MMP-9 IHC expression levels and died 2 months after treatment commenced. Patients 3 and 4 were both diagnosed with stage III adenocarcinoma. Patient 3, who had high PAI-2 and low MMP-9 IHC expression levels, was alive and had no tumor recurrence at the final follow up; patient 4, who had low PAI-2 and high MMP-9 IHC expression levels, experienced tumor recurrence within 9 months and died 14 months after treatment commenced. Photographs were taken at a magnification of 400×. Scale bars represent 100 μm. (C) Kaplan-Meier plots and ROC curve of overall survival in validation cohort showed that patients with low PAI-2 IHC expression level (*P* = 0.002) or high MMP-9 IHC expression level (*P* = 0.035) had poor overall survival. When stratified by PAI-2 and MMP-9 IHC panel, high risk group patients had low PAI-2 and high MMP-9 IHC expression levels and low risk group patients had high PAI-2 and low MMP-9 IHC expression level (*P* < 0.001). Increase of area under curve (AUC) of ROC was observed after combining PAI-2 and MMP-9 as a panel. The heat map showed that patients who were dead at the end-point tended to have low PAI-2 and high MMP-9 IHC expression level.

Based on the results that PAI-2 had the greatest prognostic value and that MMP-9 was the second most powerful prognostic marker in the multivariate analysis, a prognostic model analysis, survival analysis, and ROC curve for testing the area under the curve (AUC), sensitivity, and specificity were performed to examine the appropriateness of combining PAI-2 and MMP-9 as an IHC panel. In the prognostic model analysis (S3 Table), adding PAI-2 and MMP-9 to the pathological stage variable resulted in the highest predictive power for DFS, yet adding more markers did not further improve the predictive power. Through combining PAI-2 and MMP-9 as an IHC panel, the patients could be separated into three groups. Patients with high PAI-2 and low MMP-9 expression levels had the most favorable prognosis, and patients with low PAI-2 and high MMP-9 expression levels had the most unfavorable prognosis (*P* < 0.001 for both OS and DFS) (Fig 2A). In the ROC curve, increase of area under curve (AUC) was seen in disease-free survival after combining PAI-2 and MMP-9 as a panel (Fig 2A and S4 Table). The heat map displayed the IHC expression levels of PAI-2 and MMP-9 in individual patients clustered by end-point survival status (Fig 2A). Patients who were dead at the end-point tended to have low PAI-2 and high MMP-9 IHC expression levels. Furthermore, the cumulative prognostic effect of PAI-2 and MMP-9 was also investigated. Compared to the most favorable prognostic group (high PAI-2 and low MMP-9 IHC expression levels), the most unfavorable prognostic group (low PAI-2 and high MMP-9 IHC expression levels) showed a 6.40-fold (95% CI = 2.64–15.52; *P* < 0.001) increased risk of a poor prognosis after adjustment for pathological stage (Table 3). Fig 2B show representative examples of the usage of the PAI-2 and MMP-9 IHC panel in NSCLC samples. Patient 1, who had stage I adenocarcinoma with high PAI-2 and low MMP-9 IHC expression levels, was alive at the final follow up. In contrast, patient 2 had stage IV adenocarcinoma with low PAI-2 and high MMP-9 IHC expression levels and died 2 months after treatment commenced. Patient 3 and patient 4 were both diagnosed with stage III adenocarcinoma. Patient 3 had high PAI-2 and low MMP-9 IHC expression levels and was alive and had no tumor recurrence at the final follow up. In contrast, patient 4, who had low PAI-2 and high MMP-9 IHC expression levels, experienced tumor recurrence within 9 months and died 14 months after treatment commenced.

**Table 3:** Cox univariate and multivariate analyses of disease-free survival in association with PAI-2 and MMP-9 IHC expression levels and pathological stage in derivation cohort with 98 NSCLC cases.

Disease-free survival				
Variable	Crude HR (95%CI)	*P*	Adjusted HR (95%CI)	*P*
PAI-2 + MMP-9				
PAI-2 high & MMP-9 low	1		1	
PAI-2 high & MMP-9 high	2.78 (1.17–6.63)	0.021	3.28 (1.34–8.01)	0.009
PAI-2 low & MMP-9 low	5.08 (1.92–13.40)	0.001	4.61 (1.72–12.38)	0.002
PAI-2 low & MMP-9 high	5.74 (2.44–13.48)	<0.001	6.40 (2.64–15.52)	<0.001
Pathological stage				
I-II	-	-	1	
III-IV	-	-	3.53 (1.99–5.66)	<0.001

PAI-1: plasminogen activator inhibitor-1; PAI-2: plasminogen activator inhibitor-2; uPA: urokinase-type plasminogen activator; uPAR: urokinase-type plasminogen activator receptor; MMP-2: matrix metalloproteinase 2; MMP-9: matrix metalloproteinase 9.

### Confirmation of the prognostic value of the IHC panel comprising PAI-2 and MMP-9 in a validation cohort

To validate the prognostic power of the IHC panel comprising PAI-2 and MMP-9 discovered in our derivation cohort, we further conducted the survival analysis and ROC curve analysis in a validation cohort with 91 NSCLC patients. In Kaplan-Meier survival analysis, patients with low PAI-2 IHC expression level had a poor prognosis, and patients with high MMP-9 IHC expression level had a poor prognosis (Fig 2C). When combining PAI-2 and MMP-9 as an IHC panel, patients with high PAI-2 and low MMP-9 IHC expression levels had the best prognosis, and patients with low PAI-2 and high MMP-9 IHC expression levels had the worst outcome (*P* = 0.001). Increase of area under curve (AUC) of ROC was also noted after combining PAI-2 and MMP-9 as a panel (Fig 2C and S4 Table). As shown in the heat map (Fig 2C), patients who were dead at the end-point tended to have low PAI-2 and high MMP-9 IHC expression levels. Therefore, the PAI-2 and MMP-9 IHC panel we identified from markers of PA and MMP families could be used to stratify lung cancer patients by risk and select patients for more aggressive treatment.

## Discussion

To our knowledge, this is the first study to integrate the PA and MMP families using IHC analysis in lung cancer. We provide a novel insight that PAI-2 is the most powerful prognostic indicator among markers in PA and MMP families and, more importantly, PAI-2 remained its leading prognostic role even after taking its close relationship with other PA and MMP family markers into consideration. Research on both PA and MMP families revealed that they are an appropriate model for examining prognostic value by multi-marker assessment (S5 Table) [26–29]. With regard to the interconnected signaling pathway level, a recent study provides information about the respective contributions of each PA family member in airway epithelial cells [31]. Membrane-bound uPAR (muPAR) has been considered the key molecule because it is the only member of the PA family shown to accelerate cell migration *in vitro*. In comparison, our IHC data demonstrated no correlation between uPAR IHC expression level and patient prognosis. This discrepancy may result from several factors. First, the limitation of the previous study was that the overexpression of each marker may not accurately mimic the coordinated signaling network *in vivo* [31]. Although our data may not be able to demonstrate the cause and effect relationship between these markers, they do directly reflect the prognostic role of each marker in clinical patients in whom these markers are coexpressed and interact with each other. Moreover, the expression of ECM degradation-related enzymes, such as the PA and MMP families, may also be regulated by tumor-associated stromal cells [32]. Using a tissue microarray (TMA), we demonstrated the predictive value of these markers by considering both the tumor cells and their microenvironment. Detecting PA family markers level from tissue extraction by enzyme-linked immunosorbent assay (ELISA) was also a frequently used method to analyze their roles in lung cancer (S6 Table) [33–37]. Although ELISA could more accurately quantify the expression levels of these markers, the tumor samples may not be completely composed of tumor tissue due to not being able to see the morphology of the tissue. In contrast, IHC analysis used in present study could more avoid this limitation by directly observe the expression in situ. This may explain that the prognostic significance of PA family markers was not seen in previous study by using ELISA [33], but was seen in our study by using IHC analysis. Therefore, our results provide a complementary illustration of the roles of PA and MMP family members in lung cancer.

Our data reveal that PAI-2 plays a leading role in PAI-1, PAI-2, uPA, uPAR, MMP-2, and MMP-9. However, few studies have focused on the pathophysiological function of PAI-2 in cancer. PAI-2 overexpression was reported to reduce metastasis in xenograft models and decrease the level of uPA and migration *in vitro* [38,39]. It has recently been demonstrated that the low binding affinity of PAI-2 for endocytic receptors facilitates the clearance of uPA without evoking downstream signaling events, providing a possible explanation for the inhibitory role of PAI-2 in cancer progression [28,40]. The physiological function of PAI-2 may further provide clues regarding its role in cancer progression. As a protein expressed in stress condition such as inflammation and infection [41], elevated PAI-2 plasma level was found in patients with sepsis or leukemia, and the expression level was increased during active or relapse disease and was undetectable in remission [42,43]. In present study, we also analyzed the PAI-2 plasma level in NSCLC lung cancer patients through ELISA (S5 Fig). The finding showed elevated PAI-2 plasma level in lung cancer patients compared to normal control. Interestingly, a recent research revealed that PAI-2 which is able to inhibit uPA-mediated tumor cell migration and invasion is secreted by tumor cells itself on microparticles, not by host cells [44]. Taken together, a mechanism may be proposed that the elevated PAI-2 plasma level in lung cancer patients reflects PAI-2 positive microparticles in circulation secreted by cancer cells. And further research is clearly required to determine whether PAI-2 plasma level could be used as a surrogate for disease status in lung cancer patients.

Although the functional role of PAI-2 remains to be fully understood, by taking advantage of its inhibitory function, PAI-2 has been investigated as a vector targeting uPA in targeted alpha radioimmunotherapy for various cancer types [45]. In our study, evaluation of the expression level of PA and MMP family members by IHC analysis demonstrated provided a potential method to select patients with high uPA expression level and low PAI-2 expression level, who may obtain a greater benefit from PAI-2-targeted therapy. Moreover, the failure of broad-spectrum MMP inhibitors in clinical trials of cancer treatment has led to the development of selective MMP inhibitors [46], and inhibitors that target MMP-9 have been indicated as potential treatments for cancer [47]. Consequently, the clinical utility of PAI-2 and MMP-9 as prognostic markers identified in our study further suggests their potential for therapeutic application in NSCLC.

In conclusion, considering markers from PA and MMP protease families that is critical in regulating tumor progression, our study reveals the greatest prognostic significance of PAI-2 among uPA, uPAR, PAI-1 and PAI-2 in the PA family and MMP-2 and MMP-9 in the MMP family. By prioritizing the prognostic power of each marker, we generated an IHC panel composed of PAI-2 and MMP-9, which provides greater prognostic value than the pathological stage or a single marker alone. Integration of the prognostic values of the markers from protease families determined in the present study will allow their application in the clinical setting for predicting patient outcome and determining treatment direction in lung cancer.

## Supporting Information

S1 Materials and Methods ELISAs. Plasma levels of PAI-2 and MMP-9 were detected by commercial PAI-2 (LS-F5568, Lifespan Biosciences, USA) and MMP-9 (ab100610, Abcam, Cambridge, UK) ELISA kit. The plasma samples of 36 NSCLC patients and 6 normal controls were obtained from Kaohsiung Medical University Hospital of Taiwan.
(DOCX)

S1 Fig The PA family and MMP family interact with each other and form a complex network that regulates ECM degradation. (A and B) Both PAI-1 and PAI-2 inhibit the proteolytic ability of uPA and in turn inhibit the formation of plasmin, which can degrade the ECM alone or through the activation of MMP-2 and MMP-9. Moreover, MMP-9 can enhance uPA activity by degrading protease nexin-1. (A) The uPA-PAI-1 complex can further increase the expression of MMP-2 and MMP-9 through downstream signaling. (B) However, the uPA-PAI-2 complex facilitates the clearance of uPA without increasing MMP-2 and MMP-9 expression through downstream signaling.
(TIF)

S2 Fig Representative images of isotype negative control staining on our tissue microarray. (A) There is no IHC staining on normal lung tissue. Only non-specific staining was seen on macrophages. (B) There is no IHC staining on lung cancer cells. Only non-specific staining was seen on debrides. Photographs were taken at a magnification of 200×. Scale bars represent 200 μm.
(TIF)

S3 Fig Representative images of PAI-1, PAI-2, uPA, uPAR, MMP-2, and MMP-9 immunoexpression in lung cancer patients. Representative images showing the intensity of immunostaining for PAI-1, PAI-2, uPA, uPAR, MMP-2, and MMP-9 in lung cancer tissue microarrays. The images were taken at a magnification of 400×. Scale bars represent 200 μm.
(TIF)

S4 Fig The heat map with IHC expression level of individual patient in derivation cohort clustered by stage. Patients with higher stage tend to have low PAI-2 and high MMP-9 IHC expression levels.
(TIF)

S5 Fig Plasma level of PAI-2 and MMP-9 of NSCLC patients and normal controls. Significant elevated plasma level of PAI-2 and MMP-9 were seen in NSCLC patients compared to normal controls.
(TIF)

S1 Table Clinicopathological and demographic characteristics of derivation and validation cohort lung cancer patients. (DOCX)

S2 Table Clinicopathological analysis of the correlation between clinicopathological features and PAI-1, PAI-2, uPA, uPAR, MMP-2 and MMP-9 IHC expression in derivation cohort with 98 NSCLC cases. (DOC)

S3 Table Prognostic model analysis evaluates prognostic values of PAI-1, PAI-2, uPA, uPAR, MMP-2 and MMP-9 as IHC panels added to pathological stage in derivation cohort with 98 NSCLC cases. (DOC)

S4 Table The AUR (area under curve) of ROC (receiver operating characteristic) curve, sensitivity, and specificity of the combined and separated PAI-2 and MMP-9 IHC expression in derivation and validation cohort. (DOCX)

S5 Table Comparison of published clinicopathological studies of plasminogen activator family and matrix metalloproteinase family via immunohistochemical analysis in lung cancer. (DOCX)

S6 Table Published clinicopathological studies of plasminogen activator family markers analyzed by enzyme-linked immunosorbent assay (ELISA) in lung cancer. (DOCX)
