# Supplementary material for: Plasminogen Activator Inhibitor-2 Plays a Leading Prognostic Role among Protease Families in Non-Small Cell Lung Cancer
Source: PLoS One. 2015 Jul 31;10(7):e0133411. doi: 10.1371/journal.pone.0133411 (PMC4521958; doi:10.1371/journal.pone.0133411)
Supplement: S1 Materials and Methods — Plasma levels of PAI-2 and MMP-9 were detected by commercial PAI-2 (LS-F5568, Lifespan Biosciences, USA) and MMP-9 (ab100610, Abcam, Cambridge, UK) ELISA kit. The plasma samples of 36 NSCLC patients and 6 normal controls were obtained from Kaohsiung Medical University Hospital of Taiwan. (DOCX) [file pone.0133411.s001.docx]

**ELISAs**

Plasma levels of PAI-2 and MMP-9 were detected by commercial PAI-2 (LS-F5568, Lifespan Biosciences, USA) and MMP-9 (ab100610, Abcam, Cambridge, UK) ELISA kit. The plasma samples of 36 NSCLC patients and 6 normal controls were obtained from Kaohsiung Medical University Hospital of Taiwan.
