# Supplementary figures and images for: Plasminogen Activator Inhibitor-2 Plays a Leading Prognostic Role among Protease Families in Non-Small Cell Lung Cancer
Source: PLoS One. 2015 Jul 31;10(7):e0133411. doi: 10.1371/journal.pone.0133411 (PMC4521958; doi:10.1371/journal.pone.0133411)

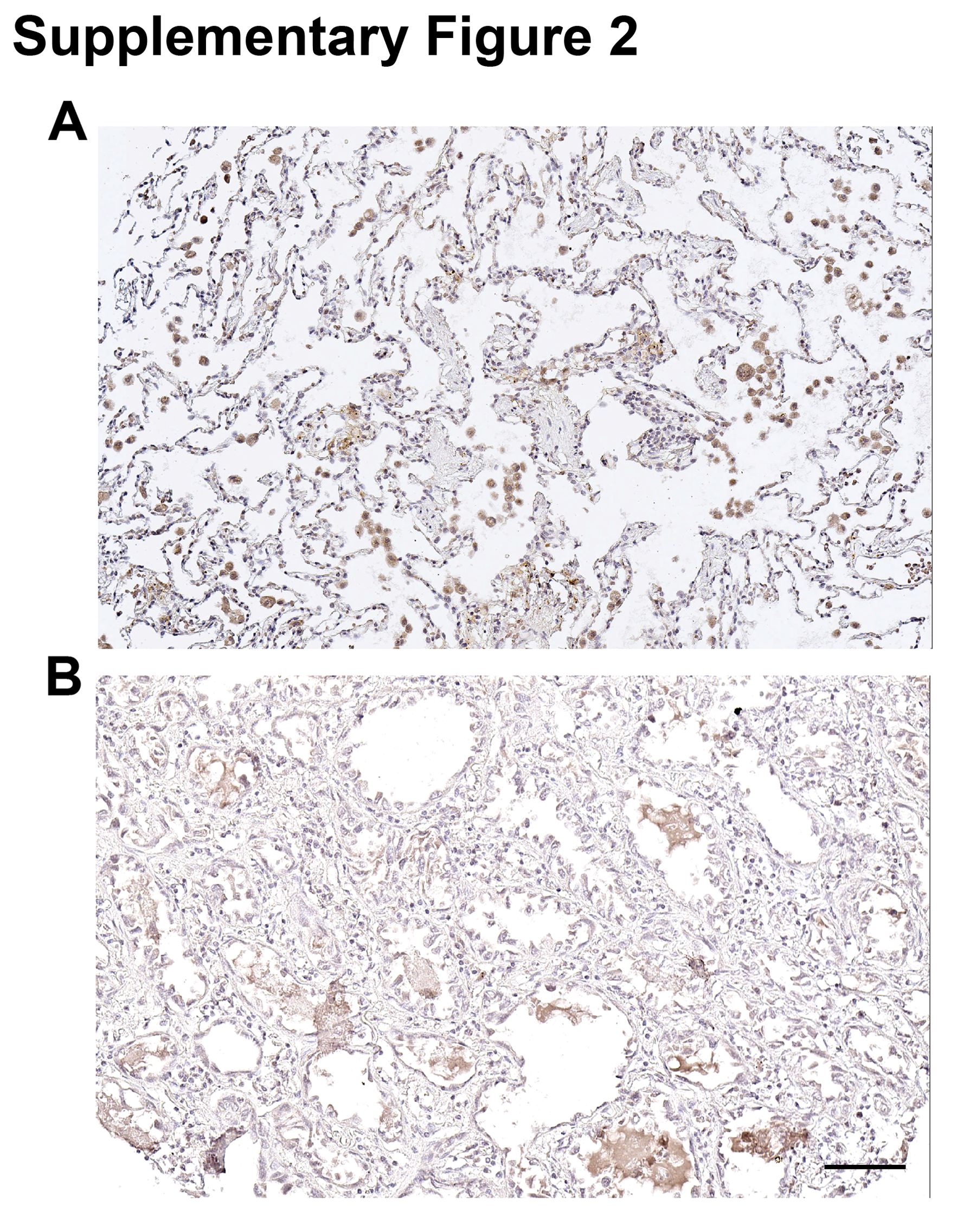

Supplement: S2 Fig — (A) There is no IHC staining on normal lung tissue. Only non-specific staining was seen on macrophages. (B) There is no IHC staining on lung cancer cells. Only non-specific staining was seen on debrides. Photographs were taken at a magnification of 200×. Scale bars represent 200 μm. (TIF) [file pone.0133411.s003.tif]

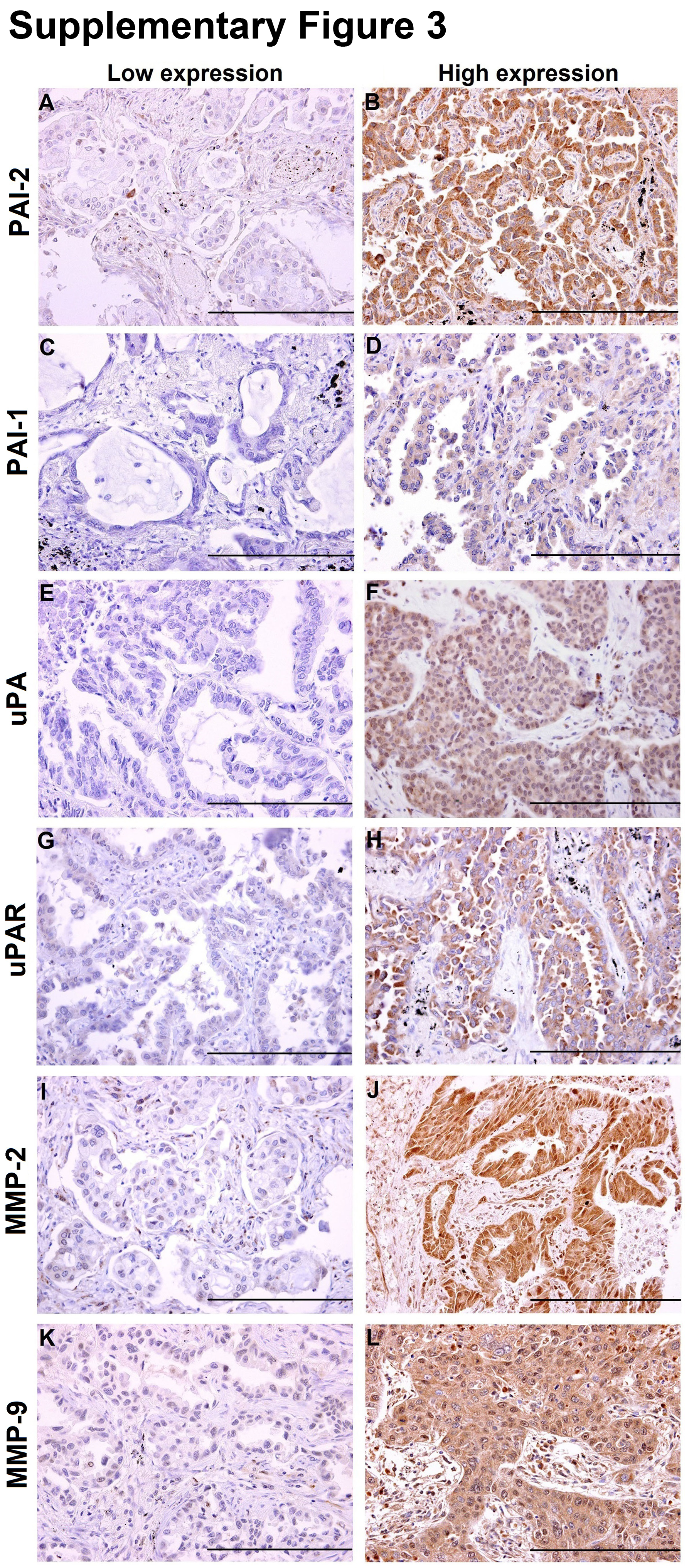

Supplement: S3 Fig — Representative images showing the intensity of immunostaining for PAI-1, PAI-2, uPA, uPAR, MMP-2, and MMP-9 in lung cancer tissue microarrays. The images were taken at a magnification of 400×. Scale bars represent 200 μm. (TIF) [file pone.0133411.s004.tif]

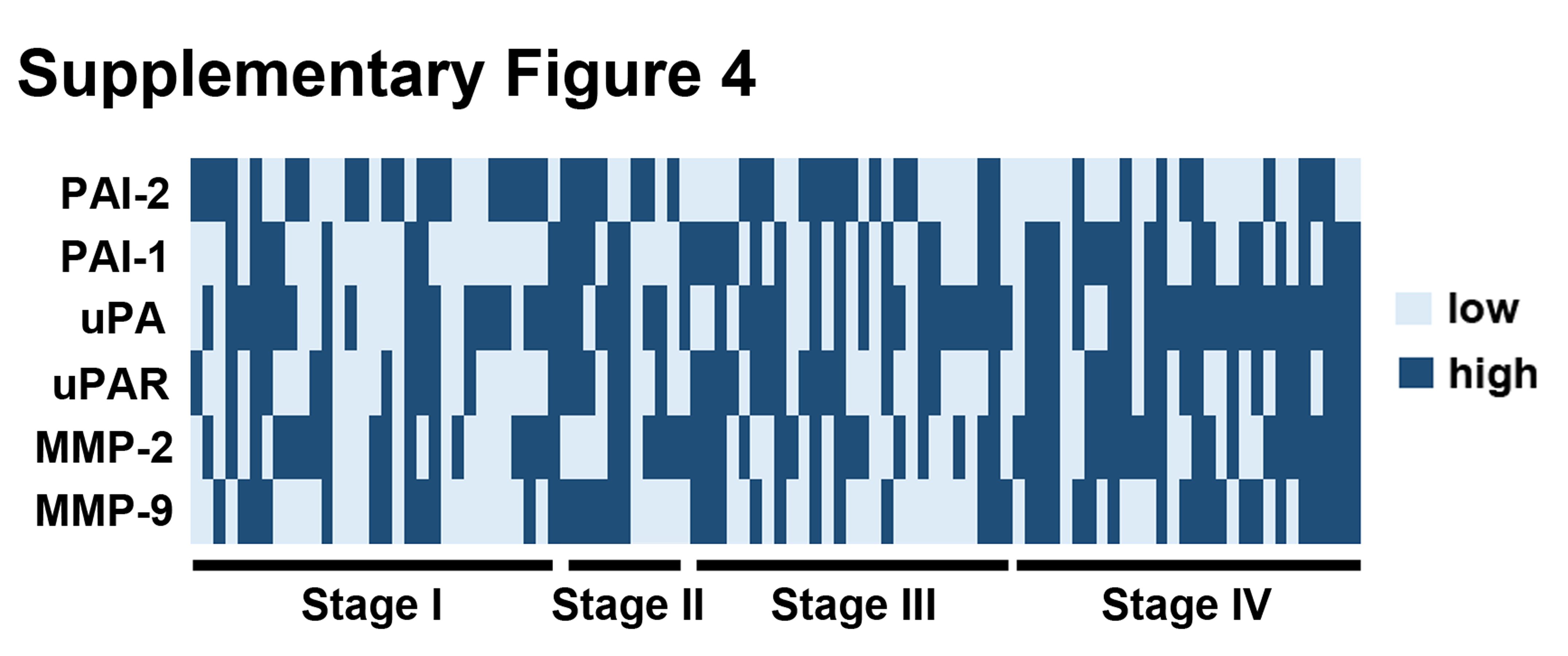

Supplement: S4 Fig — Patients with higher stage tend to have low PAI-2 and high MMP-9 IHC expression levels. (TIF) [file pone.0133411.s005.tif]

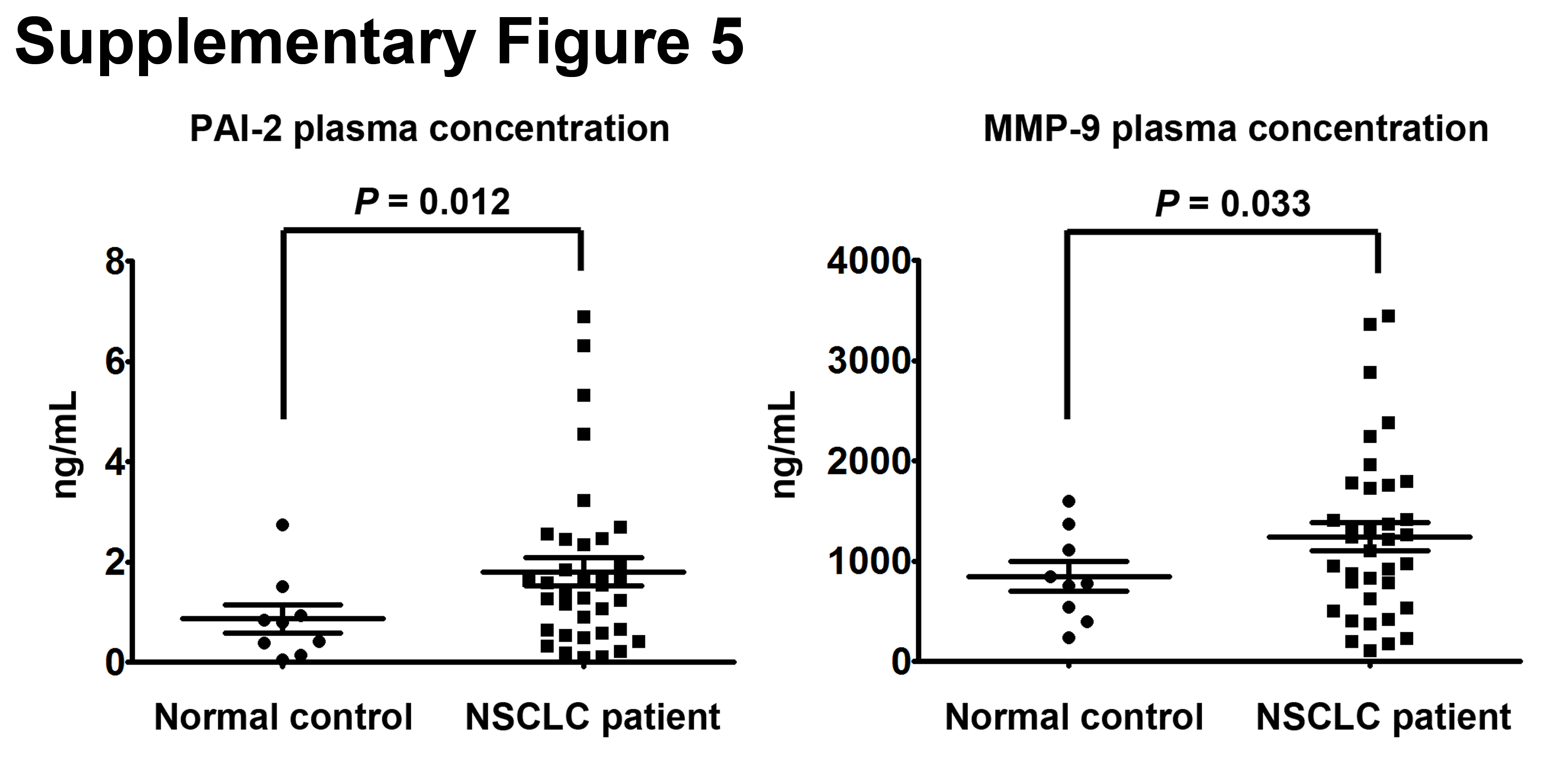

Supplement: S5 Fig — Significant elevated plasma level of PAI-2 and MMP-9 were seen in NSCLC patients compared to normal controls. (TIF) [file pone.0133411.s006.tif]
