# Supplementary material for: Plasminogen Activator Inhibitor-2 Plays a Leading Prognostic Role among Protease Families in Non-Small Cell Lung Cancer
Source: PLoS One. 2015 Jul 31;10(7):e0133411. doi: 10.1371/journal.pone.0133411 (PMC4521958; doi:10.1371/journal.pone.0133411)
Supplement: S1 Table — (DOCX) [file pone.0133411.s007.docx]

|  | Derivation cohort | Validation cohort |
| --- | --- | --- |
| Clinicopathological feature | N (%) | N (%) |
| All patients | 98 (100) | 91 |
| Age |  |  |
| <65 y | 54 (55.1) | 65 (71.4) |
| ≧65 y | 44 (44.9) | 26 (28.6) |
| Gender |  |  |
| Male | 53 (54.1) | 72 (79.1) |
| Female | 45 (45.9) | 19 (20.9) |
| Smoking |  |  |
| Smoker | 37 (37.8) | N/A |
| Non-smoker | 61 (62.2) | N/A |
| Histology subtype |  |  |
| Adenocarcinoma | 61 (62.3) | 26 (28.6) |
| Squamous cell carcinoma | 31 (31.6) | 49 (53.8) |
| Large cell carcinoma | 6 (6.1) | 8 (8.8) |
| Adenosquamous carcinoma | 0 (0) | 3 (3.3) |
| Adenoid cystic carcinoma | 0 (0) | 1 (1.1) |
| Mucoepidermoid carcinoma | 0 (0) | 4 (4.4) |
| T stage |  |  |
| T1 | 22 (22.4) | 8 (8.8) |
| T2 | 46 (46.9) | 67 (73.6) |
| T3 | 6 (6.1) | 10 (11.0) |
| T4 | 24 (24.6) | 6 (6.6) |
| N stage |  |  |
| N0 | 37 (37.8) | 50 (54.9) |
| N1 | 19 (19.4) | 23 (25.3) |
| N2 | 13 (13.2) | 18 (19.8) |
| N3 | 29 (29.6) | 0 (0) |
| M stage |  |  |
| M0 | 70 (71.4) | 90 (98.9) |
| M1 | 28 (28.6) | 1 (1.1) |
| Stage |  |  |
| I | 31 (31.6) | 34 (37.3) |
| II | 11 (11.2) | 35 (38.5) |
| III | 27 (27.6) | 21 (23.1) |
| IV | 29 (29.6) | 1 (1.1) |

**Supplementary Table 1. Clinicopathological and demographic characteristics of derivation and validation cohort lung cancer patients**
