# Supplementary material for: Plasminogen Activator Inhibitor-2 Plays a Leading Prognostic Role among Protease Families in Non-Small Cell Lung Cancer
Source: PLoS One. 2015 Jul 31;10(7):e0133411. doi: 10.1371/journal.pone.0133411 (PMC4521958; doi:10.1371/journal.pone.0133411)
Supplement: S2 Table — (DOC) [file pone.0133411.s008.doc]

**Supplementary Table 2.**

**Clinicopathological analysis of the correlation between clinicopathological features and PAI-1, PAI-2, uPA, uPAR, MMP-2 and MMP-9 IHC expression in derivation cohort with 98 NSCLC cases**

| Clinicopathological feature | n | PAI-2 expression,  *N* (%) | | PAI-1 expression,  *N* (%) | | uPA expression,  *N* (%) | | uPAR expression,  *N* (%) | | MMP-2 expression,  *N* (%) | | MMP-9 expression,  *N* (%) | |
| --- | --- | --- | --- | --- | --- | --- | --- | --- | --- | --- | --- | --- | --- |
|  |  | Low (*N* =48) | High (*N* =50) | Low (*N* =46) | High (*N* =52) | Low (*N* =51) | High (*N* =47) | Low (*N* =50) | High (*N* =48) | Low (*N* =38) | High (*N* =60) | Low (*N* =29) | High (*N* =69) |
| **Age** |  |  |  |  |  |  |  |  |  |  |  |  |  |
| <65 y | 54 | 29 (53.7) | 25 (46.3) | 23 (42.6) | 31 (57.4) | 26 (48.1) | 28 (51.9) | 24 (44.4) | 30 (55.6) | 22 (40.7) | 32 (59.3) | 15 (27.8) | 39 (72.2) |
| ≧65 y | 44 | 21 (47.7) | 23 (52.3) | 23 (52.3) | 21 (47.7) | 25 (56.8) | 19 (43.2) | 26 (59.1) | 18 (40.9) | 16 (36.4) | 28 (63.6) | 14 (31.8) | 30 (68.2) |
| *P* value |  | 0.556 | | 0.340 | | 0.393 | | 0.149 | | 0.658 | | 0.663 | |
| **Gender** |  |  |  |  |  |  |  |  |  |  |  |  |  |
| Male | 53 | 26 (49.1) | 27 (50.9) | 26 (49.1) | 27 (50.9) | 30 (56.6) | 23 (43.4) | 27 (50.9) | 26 (49.1) | 24 (45.3) | 29 (54.7) | 12 (22.6) | 41 (77.4) |
| Female | 45 | 24 (53.3) | 21 (46.7) | 20 (44.4) | 25 (55.6) | 21 (46.7) | 24 (53.3) | 23 (51.1) | 22 (48.9) | 14 (31.1) | 31 (68.9) | 17 (37.8) | 28 (62.2) |
| *P* value |  | 0.673 | | 0.648 | | 0.218 | | 0.987 | | 0.151 | | 0.102 | |
| **Smoking** |  |  |  |  |  |  |  |  |  |  |  |  |  |
| Smoker | 37 | 23 (62.2) | 14 (37.8) | 16 (43.2) | 21 (56.8) | 20 (54.1) | 17 (45.9) | 17 (45.9) | 20 (54.1) | 17 (45.9) | 20 (54.1) | 12 (32.4) | 25 (67.6) |
| Non-smoker | 61 | 27 (44.3) | 34 (55.7) | 30 (49.2) | 31 (50.8) | 31 (50.8) | 30 (49.2) | 33 (54.1) | 28 (45.9) | 21 (34.4) | 40 (65.6) | 17 (27.9) | 44 (72.1) |
| *P* value |  | 0.086 | | 0.568 | | 0.756 | | 0.434 | | 0.257 | | 0.631 | |
| **Histology** |  |  |  |  |  |  |  |  |  |  |  |  |  |
| Adenocarcinoma | 61 | 30 (49.2) | 31 (50.8) | 23 (37.7) | 38 (62.3) | 27 (44.3) | 34 (55.7) | 27 (44.3) | 34 (55.7) | 24 (39.3) | 37 (60.7) | 23 (37.7) | 38 (62.3) |
| SCC | 31 | 15 (48.4) | 15 (49.0) | 21 (67.7) | 10 (32.3) | 22 (71.0) | 9 (29.0) | 20 (64.5) | 11 (35.5) | 13 (41.9) | 18 (58.1) | 6 (19.4) | 25 (80.6) |
| LCC | 6 | 5 (83.3) | 1 (16.7) | 2 (33.3) | 4 (66.7) | 2 (33.3) | 4 (66.7) | 3 (50.0) | 3 (50.0) | 1 (16.7) | 5 (83.3) | 0 (0) | 6 (100) |
| *P* value |  | 0.262 | | 0.019 | | 0.034 | | 0.185 | | 0.503 | | 0.050 | |
| **T status** |  |  |  |  |  |  |  |  |  |  |  |  |  |
| T1+T2 | 68 | 34 (50.0) | 34 (50.0) | 29 (42.6) | 39 (57.4) | 31 (45.6) | 37 (54.4) | 32 (47.1) | 36 (52.9) | 26 (38.2) | 42 (61.8) | 18 (26.5) | 50 (73.5) |
| T3+T4 | 30 | 16 (53.3) | 14 (46.7) | 17 (56.7) | 13 (43.3) | 20 (66.7) | 10 (33.3) | 18 (60.0) | 12 (40.0) | 12 (40.0) | 18 (60.0) | 11 (36.7) | 19 (63.3) |
| *P* value |  | 0.761 | | 0.200 | | 0.054 | | 0.238 | | 0.869 | | 0.308 | |
| **N status** |  |  |  |  |  |  |  |  |  |  |  |  |  |
| N0 | 37 | 16 (43.2) | 21 (56.8) | 22 (59.5) | 15 (40.5) | 25 (67.6) | 12 (32.4) | 21 (56.8) | 16 (43.2) | 15 (40.5) | 22 (59.5) | 14 (37.8) | 23 (62.2) |
| N1-3 | 61 | 34 (55.7) | 27 (44.3) | 24 (39.3) | 37 (60.7) | 26 (42.6) | 35 (57.4) | 29 (47.5) | 32 (52.5) | 23 (37.7) | 38 (62.3) | 15 (24.6) | 46 (75.4) |
| *P* value |  | 0.230 | | 0.053 | | 0.014 | | 0.376 | | 0.780 | | 0.164 | |
| **M status** |  |  |  |  |  |  |  |  |  |  |  |  |  |
| M0 | 70 | 31 (44.3) | 39 (55.7) | 35 (50.0) | 35 (50.0) | 39 (55.7) | 31 (44.3) | 40 (57.1) | 30 (42.9) | 30 (42.9) | 40 (57.1) | 24 (34.3) | 46 (65.7) |
| M1 | 28 | 19 (67.9) | 9 (32.1) | 11 (39.3) | 17 (60.7) | 12 (42.9) | 16 (57.1) | 10 (35.7) | 18 (64.3) | 8 (28.6) | 20 (71.4) | 5 (17.9) | 23 (82.1) |
| *P* value |  | 0.035 | | 0.337 | | 0.250 | | 0.055 | | 0.190 | | 0.107 | |
| **Pathological stage** |  |  |  |  |  |  |  |  |  |  |  |  |  |
| I + II | 42 | 16 (38.1) | 26 (61.9) | 24 (57.1) | 18 (42.9) | 28 (66.7) | 14 (33.3) | 24 (57.1) | 18 (42.9) | 19 (45.2) | 23 (54.8) | 15 (35.7) | 27 (64.3) |
| III + IV | 56 | 34 (60.7) | 22 (39.3) | 22 (39.3) | 34 (60.7) | 23 (41.1) | 33 (58.9) | 26 (46.4) | 30 (53.6) | 19 (33.9) | 37 (66.1) | 14 (25.0) | 42 (75.0) |
| *P* value |  | 0.027 | | 0.080 | | 0.012 | | 0.294 | | 0.255 | | 0.250 | |
| **Recurrence** |  |  |  |  |  |  |  |  |  |  |  |  |  |
| No | 53 | 21 (39.6) | 32 (60.4) | 29 (54.7) | 24 (45.3) | 39 (76.5) | 12 (23.5) | 34 (68.0) | 16 (32.0) | 22 (57.9) | 16 (42.1) | 18 (62.1) | 11 (37.9) |
| Yes | 45 | 29 (64.4) | 16 (35.6) | 17 (37.8) | 28 (62.2) | 7 (14.9) | 40 (85.1) | 12 (25.0) | 36 (75.0) | 24 (40.0) | 36 (60.0) | 28 (40.6) | 41 (59.4) |
| *P* value |  | 0.014 | | 0.094 | | < 0.001 | | < 0.001 |  | 0.084 | | 0.052 | |
