# Supplementary material for: Plasminogen Activator Inhibitor-2 Plays a Leading Prognostic Role among Protease Families in Non-Small Cell Lung Cancer
Source: PLoS One. 2015 Jul 31;10(7):e0133411. doi: 10.1371/journal.pone.0133411 (PMC4521958; doi:10.1371/journal.pone.0133411)
Supplement: S3 Table — (DOC) [file pone.0133411.s009.doc]

**Supplementary Table 3. Prognostic model analysis evaluates prognostic values of PAI-1, PAI-2, uPA, uPAR, MMP-2 and MMP-9 as IHC panels added to pathological stage in derivation cohort with 98 NSCLC cases**

| Disease-free survival |  |  |
| --- | --- | --- |
| Model | Chi-Square statistic | *P* |
| Stage only (reference) ^a^ | - | - |
| Stage + PAI-2 | 13.995 | <0.001 |
| Stage + PAI-2 + MMP-9 | 6.481 | 0.011 |
| Stage + PAI-2 + MMP-9 + PAI-1 | 3.388 | 0.066 |
| Stage + PAI-2 + MMP-9 + PAI-1 + uPAR | 2.319 | 0.128 |
| Stage + PAI-2 + MMP-9 + PAI-1 + uPAR + MMP-2 | 0.008 | 0.766 |
| Stage + PAI-2 + MMP-9 + PAI-1 + uPAR + MMP-2 + uPA | 0.030 | 0.863 |
| ^a^ Pathological stage I-II vs. III-IV |  |  |
