# Supplementary material for: Plasminogen Activator Inhibitor-2 Plays a Leading Prognostic Role among Protease Families in Non-Small Cell Lung Cancer
Source: PLoS One. 2015 Jul 31;10(7):e0133411. doi: 10.1371/journal.pone.0133411 (PMC4521958; doi:10.1371/journal.pone.0133411)
Supplement: S4 Table — (DOCX) [file pone.0133411.s010.docx]

| **Derivation cohort (Overall survival)** | AUC | Sensitivity | | | Specificity | |  |
| --- | --- | --- | --- | --- | --- | --- | --- |
| PAI2-2 + MMP-9 | 0.663 | PAI-2 high/MMP-9 low vs. all others | 90.1% | | | 33.3% | |
|  |  | PAI-2 low/MMP-9 high vs. all others | 43.7% | | | 77.8% | |
| PAI-2 | 0.673 | 60.6% | | | 74.1% | |  |
| MMP-9 | 0.551 | 73.2% | | | 37.0% | |  |
| **Derivation cohort (Disease-free survival)** | AUC | Sensitivity | | | Specificity | |  |
| PAI2-2 + MMP-9 | 0.704 | PAI-2 high/MMP-9 low vs. all others | | 90.7% | 39.1% | |  |
|  |  | PAI-2 low/MMP-9 high vs. all others | | 44.0% | 82.6% | |  |
| PAI-2 | 0.691 | 60.0% | | | 78.3% | |  |
| MMP-9 | 0.591 | 74.7% | | | 43.5% | |  |
| **Validation cohort (overall survival)** | AUC | Sensitivity | | | Specificity | |  |
| PAI2-2 + MMP-9 | 0.720 | PAI-2 high/MMP-9 low vs. all others | 96.2% | | | 33.3% |  |
|  |  | PAI-2 low/MMP-9 high vs. all others | 36.5% | | | 66.7% |  |
| PAI-2 | 0.670 | 67.3% | | | 66.7% | |  |
| MMP-9 | 0.612 | 63.5% | | | 59.0% | |  |

**Supplementary Table 4.** The AUR (area under curve) of ROC (receiver operating characteristic) curve, sensitivity, and specificity of the combined and separated PAI-2 and MMP-9 IHC expression in derivation and validation cohort
