# Supplementary material for: Plasminogen Activator Inhibitor-2 Plays a Leading Prognostic Role among Protease Families in Non-Small Cell Lung Cancer
Source: PLoS One. 2015 Jul 31;10(7):e0133411. doi: 10.1371/journal.pone.0133411 (PMC4521958; doi:10.1371/journal.pone.0133411)
Supplement: S5 Table — (DOCX) [file pone.0133411.s011.docx]

### Supplementary Table 5. Comparison of published clinicopathological studies of plasminogen activator family and matrix metalloproteinase family via immunohistochemical analysis in lung cancer.

| Reference | Histology | Plasminogen activator family | | | |  | Reference | Histology | Matrix metalloproteinase family | |
| --- | --- | --- | --- | --- | --- | --- | --- | --- | --- | --- |
|  |  | uPA | uPAR | PAI-1 | PAI-2 |  |  |  | MMP-2 | MMP-9 |
| Oka *et al.*, 1991[11] | Adenocarcinoma | (-) | × | × | × |  | Qian *et al.*, 2010[14] | NSCLC  (meta-analysis) | (-) | × |
| Pavey *et al.,* 1996[12] | NSCLC | = | = | (-) | × |  | Peng *et al.*, 2012[15] | NSCLC (meta-analysis) | × | (-) |
| Yoshino *et al.,*1998[19] | NSCLC and small cell lung cancer | = | = | = | (+) |  | Cox *et al*, 2001[18] | Stage  I-IIIA  NSCLC | = | (-) |
| Volm *et al.*, 1999[13] | NSCLC | (-) | = | × | × |  | Ishikawa *et al*, 2004[21] | Stage  I-IIIA  NSCLC | (-)^a^ | = |
| Robert *et al.*, 1999[20] | NSCLC | (-)^a^ | × | (-)^b^ | (+)^a^ |  | Shao *et al.*, 2011[17] | Stage IA  NSCLC | =^c^ | (-)^c^ |
| Present study ^c^ | NSCLC | = | = | = | (+)  Prognostic significance: 1^st^ |  | Present study ^c^ | NSCLC | = | (-)  Prognostic significance: 2^nd^ |
| (-): High expression correlated with unfavorable prognosis or unfavorable clinicopathological parameters such as higher T, N and M stage.  (+): High expression correlated with favorable prognosis or favorable clinicopathological parameters such as lower T, N and M stage.  =: The expression level did not correlate with prognosis.  ×: Not included in the study  ^a^ Results from stromal cell IHC expression.  ^b^ High tumor cell PAI-1 expression correlated with nodal involvement and higher tumor stage. High fibroblastic PAI-1 expression correlated with higher tumor stage.  ^c^ From the results of multivariate cox regression analysis. | | | | | | | | | | |
