# Supplementary material for: Plasminogen Activator Inhibitor-2 Plays a Leading Prognostic Role among Protease Families in Non-Small Cell Lung Cancer
Source: PLoS One. 2015 Jul 31;10(7):e0133411. doi: 10.1371/journal.pone.0133411 (PMC4521958; doi:10.1371/journal.pone.0133411)
Supplement: S6 Table — (DOCX) [file pone.0133411.s012.docx]

### Supplementary Table 6. Published clinicopathological studies of plasminogen activator family markers from lung cancer tissue extraction analyzed by enzyme-linked immunosorbent assay (ELISA)

| Reference | Histology | Plasminogen activator family | | | |
| --- | --- | --- | --- | --- | --- |
|  |  | uPA | uPAR | PAI-1 | PAI-2 |
| Pedersen *et al.*, 1994 [34] | Adenocarcinoma | = | × | (-) | × |
| Pedersen *et al.*, 1994 [35] | SCC and LCC | = | (-) | = | × |
| Salden *et al.*, 2000 [33] | NSCLC | = * | = * | = * | = * |
| Werle *et al.*, 2004 [36] | NSCLC | × | (-) | (-) | × |
| Offersen *et al.*, 2007 [37] | NSCLC | = | × | = | × |
| (-): High expression correlated with unfavorable prognosis or unfavorable clinicopathological parameters such as higher T, N and M stage.  (+): High expression correlated with favorable prognosis or favorable clinicopathological parameters such as lower T, N and M stage.  =: The expression level did not correlate with prognosis.  ×: Not included in the study  * Higher expression levels of all four markers in plasminogen activator family were found in tumor tissue as compared to normal lung tissue, but there were no significant relationships between their level and survival. | | | | | |
